# Supplementary material for: Fasciola hepatica Fatty Acid Binding Protein 1 Modulates T cell Polarization by Promoting Dendritic Cell Thrombospondin-1 Secretion Without Affecting Metabolic Homeostasis in Obese Mice
Source: Front Immunol. 2022 May 26;13:884663. doi: 10.3389/fimmu.2022.884663 (PMC9204345; doi:10.3389/fimmu.2022.884663)
Supplement: Supplementary file 1 [file DataSheet_1.docx]

**Supplementary methods**

**Confocal imaging**

5 × 10^4^ moDC/chamber were seeded onto poly-D-lysine (Sigma-Aldrich) coated coverslips of a glass bottom dish (ø35 mm; MatTek Corporation) for 24 hours. Cells were incubated with LPS (100 ng/ml) and *fh*FABP1 (25 μg/ml) labelled with Promofluor-660 labeling kit (Promokine) for 4 hours. The nuclei of the cells were stained with Hoechst (1:250, 1 mg/ml) and the cell membrane was stained with WGA-Cy3 (1:100, 14 µM). Images were taken at room temperature on a Leica TCS (true confocal scanning) SP8 WLL (white light laser) microscope (Leica Microsystems). The sequential scanning mode was applied to image Hoechst (excitation: 405 nm; emission: 420-470 nm), WGA-Cy3 (excitation: 560 nm, emission: 570-620 nm) and PromoFluor-660-*fh*FABP1 (excitation: 647 nm; emission: 655-705 nm). For imaging the uptake of PromoFluor-660-*fh*FABP1, a 63x objective (Leica HC PL APO 63x/1.40na OIL CS2) was used. The images were generated using the Leica software (LAS X; Leica Microsystems).

**Computational analyses of RNA sequencing data**

The sequencing data quality assessment was performed using FastQC v. 0.11.9 and MultiQC v. 1.7 [1]. Trimming of low-quality sequences and adapter content was performed with cutadapt v. 2.4 [2], with the following parameters: --error-rate 0.1 --quality-cutoff 20 --minimum-length 10. The alignment of reads to the GRCh38 human reference genome has been performed using STAR v. 2.7.7a [3], using Ensembl GRCh38 version 102 genome annotation. Standard ENCODE alignment options were used, namely: --outFilterType BySJout --outFilterMultimapNmax 20 --alignSJoverhangMin 8 --alignSJDBoverhangMin 1 --outFilterMismatchNmax 999 --outFilterMismatchNoverReadLmax 0.04 --alignIntronMin 20 --alignIntronMax 1000000 --alignMatesGapMax 1000000. The duplicated alignments were flagged by the MarkDuplicates program from the Picard v.2.24.0 software suite [4]. The alignment filtering was performed with the samtools v. 1.10 software [5], with the following parameters: -q 1 -f3 -F1280. The read quantification has been performed using htseq-count script from the HTSeq v. 0.13.5 python package [6]. The filtered alignments were counted per exon on gene-level, with the default “union” counting method. The data processing pipeline was written in Nexflow [7]. The source code can be accessed from the GitLab code repository: <https://gitlab.com/lapinskim/pbmcs_seq>. Differential expression analysis for the stimulated PBMCS cells has been performed in R programming language v. 4.0.3 [8] with DESeq2 v. 1.30.0 R package [9]. The log fold change shrinkage for visualisations was performed using the apeglm algorithm [10].

**Metabolic profiling of moDCs**

For assessing their metabolic characteristics. 5 x 10^4^ moDCs stimulated with LPS (100 ng/ml) and *fh*FABP1 (25 μg/ml) for 5 hours were plated in unbuffered, glucose-free RPMI supplemented with 5% FCS and left to rest 1 hour at 37°C in an CO^2^ free incubator. Extracellular acidification rate (ECAR) and oxygen consumption rate (OCR) were next measured using a Seahorse XFe96 Extracellular Flux Analyzer (Seahorse Bioscience) before and after addition of glucose (10 mM), oligomycin (1 μM), fluoro-carbonyl cyanide phenylhydrazone (FCCP, 3 μM), and rotenone/antimycin A (1/1 μM) (all Sigma-Aldrich).

**Lipidomic analysis**

moDCs (0.5 x 10^6^/ml) were stimulated with 100 ng/ml LPS and 25 μg/ml *fh*FABP1. After 48 hours cells were harvested, washed twice with PBS and snap frozen. Lipids were extracted by the methyl-tert-butylether method and analyzed using the Lipidyzer™, a direct infusion-tandem mass spectrometry (DI-MS/MS)-based platform (Sciex, Redwood City, USA), as previously described [11]. Lipid concentrations were expressed as nmol/10^6^ cells.

**Reference**

[1] P. Ewels, M. Magnusson, S. Lundin, and M. Kaller, MultiQC: summarize analysis results for multiple tools and samples in a single report. Bioinformatics 32 (2016) 3047-8.

[2] M. Martin, Cutadapt removes adapter sequences from high-throughput sequencing reads. EMBnet.journal 17 (2011) 10-12.

[3] A. Dobin, C.A. Davis, F. Schlesinger, J. Drenkow, C. Zaleski, S. Jha, P. Batut, M. Chaisson, and T.R. Gingeras, STAR: ultrafast universal RNA-seq aligner. Bioinformatics 29 (2013) 15-21.

[4] B. Institute, Picard Toolkit, 2021.

[5] H. Li, B. Handsaker, A. Wysoker, T. Fennell, J. Ruan, N. Homer, G. Marth, G. Abecasis, R. Durbin, and S. Genome Project Data Processing, The Sequence Alignment/Map format and SAMtools. Bioinformatics 25 (2009) 2078-9.

[6] S. Anders, P.T. Pyl, and W. Huber, HTSeq--a Python framework to work with high-throughput sequencing data. Bioinformatics 31 (2015) 166-9.

[7] P. Di Tommaso, M. Chatzou, E.W. Floden, P.P. Barja, E. Palumbo, and C. Notredame, Nextflow enables reproducible computational workflows. Nat Biotechnol 35 (2017) 316-319.

[8] T. RC, R: A Language and Environment for Statistical Computing, 2019.

[9] M.I. Love, W. Huber, and S. Anders, Moderated estimation of fold change and dispersion for RNA-seq data with DESeq2. Genome Biol 15 (2014) 550.

[10] A. Zhu, J.G. Ibrahim, and M.I. Love, Heavy-tailed prior distributions for sequence count data: removing the noise and preserving large differences. Bioinformatics 35 (2019) 2084-2092.

[11] J.F. Zinsou, J.J. Janse, Y.Y. Honpkehedji, J.C. Dejon-Agobe, N. Garcia-Tardon, P.T. Hoekstra, M. Massinga-Loembe, P. Corstjens, G.J. van Dam, M. Giera, P.G. Kremsner, M. Yazdanbakhsh, A.A. Adegnika, and B. Guigas, Schistosoma haematobium infection is associated with lower serum cholesterol levels and improved lipid profile in overweight/obese individuals. PLoS Negl Trop Dis 14 (2020) e0008464.

[12] N.C. Zembruski, V. Stache, W.E. Haefeli, and J. Weiss, 7-Aminoactinomycin D for apoptosis staining in flow cytometry. Anal Biochem 429 (2012) 79-81.

**Supplementary table**

**Table S1. FACS antibodies and ELISA kits**

| **Target** | **Type** | **Source** | **Identifier** |
| --- | --- | --- | --- |
| ***Human*** |  |  |  |
| Aqua | Cell death marker | Invitrogen/Thermo | L34957 |
| 7-AAD | Cell death marker | Invitrogen/Thermo | A1310 |
| CD40 | Antibody | BD Pharmingen | 555591 |
| CD80 | Antibody | BD | 560442 |
| CD83 | Antibody | Invitrogen | 12-083942 |
| CD86 | Antibody | BD Pharmingen | 561128 |
| HLA-DR | Antibody | Invitrogen | 47-9956-42 |
| CD163 | Antibody | BioLegend | 333608 |
| CD103 | Antibody | BioLegend | 350211 |
| CD85k (ILT3) | Antibody | BioLegend | 333015 |
| CD274 (PD-L1) | Antibody | eBioscience | 12-5983 |
| IL-4 | Antibody | BD Bioscience | 554516 |
| IFNγ | Antibody | BD Bioscience | 560371 |
| IL-10 | Antibody | BD Bioscience | 554707 |
| FoxP3 | Antibody | eBioscience | 17-4776-42 |
| GATA3 | Antibody | eBioscience | 50-9966-42 |
| T-bet | Antibody | eBioscience | 45-5825 |
| CD3 | Antibody | eBioscience | 46-0036 |
| CD4 | Antibody | BD Bioscience | 557852 |
| CD25 | Antibody | BD Bioscience | 340907 |
| TSP-1 | Antibody | Merck/Sigma-Aldrich | MABT879 |
| IgG2b | Antibody | BioLegend | 400347 |
| CCL19 | Chemokine | PeproTech | 300-29B |
| CXCL9 | Chemokine | PeproTech | 300-26 |
| CXCL11 | Chemokine | PeproTech | 300-46 |
| IL-10 | ELISA kit | BD Bioscience | 555157 |
| IL-12p70 | ELISA kit | BD Bioscience | 555183 |
| IL-6 | ELISA kit | BD Bioscience | 555220 |
| TSP-1 | ELISA kit | R&D Systems | DY3074 |
| TGFβ | ELISA kit | R&D Systems | DY240 |
| CXCL11 | ELISA kit | R&D Systems | DY672 |
| ***Mouse*** |  |  |  |
| B220 | Antibody | eBioscience | 11-0452 |
| CD3 | Antibody | Biolegend | 100237 |
| CD4 | Antibody | BD | 563232 |
| CD11b | Antibody | eBioscience | 11-0112 |
| CD11c | Antibody | BD | 553801 |
| CD44 | Antibody | eBioscience | 56-0441 |
| CD44 | Antibody | eBioscience | 48-0441 |
| CD45 | Antibody | Biolegend | 103149 |
| CD64 | Antibody | Biolegend | 139304 |
| F4/80 | Antibody | Biolegend | 123147 |
| GR-1 | Antibody | BD | 553127 |
| IFNγ | Antibody | BD | 561479 |
| IFNγ | Antibody | eBioscience | 25-7311 |
| IL-4 | Antibody | eBioscience | 17-7041 |
| IL-5 | Antibody | Biolegend | 504303 |
| IL-10 | Antibody | eBioscience | 12-7101 |
| Ly6C | Antibody | Biolegend | 128025 |
| NK 1.1 | Antibody | eBioscience | 11-5941 |
| Siglec-F | Antibody | BD | 740388 |
| YM1-biotin | Antibody | R&D Systems | BAF2446 |
| Streptavidin-PerCP-Cy5.5 | Reagent | Biolegend | 405214 |
| Zombie-UV viability kit | Cell death marker | Biolegend | 423107 |

**Supplementary figures**

**Figure S1. Gating strategies.** Representative gating strategies for the identification of eosinophils, macrophages, Relmα^+^/CD11c^-^YM1^+^/macrophages, CD4^+^ T cells, IL-5^+^ Th2 cells and IFNγ^+^ Th1 cells in eWAT. A sample from the *fh*FABP1-treated group is shown as example. Gating strategies were similar for liver samples.

**Figure S2. *Fasciola hepatica* FABP1 is internalized by dendritic cells and mainly localized in the intracellular compartment.** The subcellular localization of PF-647-labeled recombinant *fh*FABP1 (25 μg/ml; depicted in red) in moDCs was determined after 4 hours by confocal microscopy and shown as 2D images of different Z-planes with nuclear (Hoechst, depicted in blue) and membrane (WGA-Cy3, depicted in yellow) staining. One representative experiment is shown from 3 independent experiments.

**Figure S3. *Fasciola hepatica* FABP1 does not induce apoptosis/cell death.** Human moDCs were treated for 48 h with increasing concentration of *fh*FABP1 (10, 25, 50 µg/ml) in presence of LPS (100 ng/ml). Viable, apoptotic and late apoptotic/dead cells were determined by flow cytometry as described in [12]. One representative experiment is shown for the Zebra plot. All data are expressed as mean ± SEM (n=4 independent experiments).

**Figure S4. *Fasciola hepatica* FABP1 induces CD103 expression in TNFα/IL-1β-primed dendritic cells and IL-10 producing T cells.** Human moDCs were treated for 48 h with or without 25 µg/ml recombinant *fh*FABP1 (orange bars) in presence of TNFα (0.05 µg/ml) and IL-1β (0.025 µg/ml) as a maturation cocktail **(a)**. The cell-surface expression of tolerogenic markers **(b)** and cytokines secretion **(c)** by moDCs were determined by flow cytometry and ELISA, respectively. Conditioned moDCs were characterized for their capacity to prime CD4+ T response **(d)** after 11 days of co-culture, as described in Figure 1. All data are expressed relative to the DCs stimulated with TNFα/IL-1β alone (dash line) as mean ± SEM. * P ≤ 0.05 *vs* LPS alone (n=3-7 independent experiments).

**Figure S5. *Fasciola hepatica* FABP1 affects mRNA and protein expression of various cytokines/chemokines in LPS-stimulated dendritic cells.** Human moDCs were stimulated for 5 h **(a)** or 48h **(b)** with PBS or various concentrations of recombinant *fh*FABP1 in presence of 100 ng/ml LPS. qPCR confirmation of RNAseq data showing changes in gene expression levels of various cytokines/chemokines in moDCs stimulated with 25 µg/ml *fh*FABP1 **(a)**. The effect of increasing concentrations (10, 25, 50 µg/ml) of *fh*FABP1 on TSP-1 secretion by moDCs was determined by ELISA **(b)**. All data are expressed relative to the DCs stimulated with LPS alone (dash line) as mean ± SEM. * P ≤ 0.05 *vs* LPS alone (n=3-5 independent experiments).

**Figure S6. *Fasciola hepatica* FABP1 does not affect intrinsic metabolism of dendritic cells**

Human moDCs were stimulated for 5 h with PBS (black squares/bars) or 25 µg/ml recombinant *fh*FABP1 (orange squares/bars) in presence of 100 ng/ml LPS. Gene set enrichment analyses were done on the RNAseq data from Figure 5 using the Reactome module for the main metabolic pathways **(a)**. The intrinsic metabolic phenotype of moDCs were determined by measuring extracellular acidification rates (ECAR) and oxygen consumption rates (OCR) with a Seahorse flux analyser **(c)**. The mitochondrial mass **(b)** and intracellular neutral lipid content **(d)** were determined by flow cytometry, using Mitotracker green and Bodipy staining, respectively. The lipid class concentrations were also quantified by targeted lipidomics using the Lipidyzer platform **(e)**. PC, Phosphatidylcholine; PE, Phosphatidylethanolamine; LPC, Lysophosphatidylcholine; LPE, Lysophosphatidylethanolamine; SM, Sphingomyelin; CER, Ceramides; CE, Cholesterylester; DG, Diglycerides; TG, Triglycerides; FFA, Free-fatty acids. Results are expressed as mean ± SEM (n=3-5 independent experiments).

**Figure S7.** ***Fasciola hepatica* FABP1 does not induce type 2 immune response in livers from obese mice.** Mice were fed a high-fat diet (HFD) for a period of 12 weeks and next received intraperitoneal injections of 25 µg recombinant *fh*FABP1 (orange squares/bars) or vehicle (PBS, black squares/bars) every 3 days for 4 weeks, as described in Figure 8. The number of liver total leukocytes **(a)**, Kupffer cells **(b)** and CD4^+^ T cells **(c)**, together with the percentage of CD44^+^, IL-5^+^, IFNγ^+^ CD4^+^ T cells **(d)** were determined by flow cytometry. The IL-5^+^-on-IFNγ^+^ ratio was calculated **(e)**. Results are expressed as mean ± SEM. # P ≤ 0.05 *vs* HFD (n=5-6 mice per group).
